# A systematic review and diagnostic test accuracy meta-analysis of the validity of anion gap as a screening tool for hyperlactatemia

**DOI:** 10.1186/s13104-017-2853-9

**Published:** 2017-11-03

**Authors:** Stella Andrea Glasmacher, William Stones

**Affiliations:** 1 Brighton and Sussex Medical School, BSMS Teaching Building, University of Sussex, Brighton, BN1 9PX UK; 2 Departments of Public Health and Obstetrics & Gynaecology, Malawi College of Medicine, Private Bag 360, Chichiri, Blantyre 3, Malawi

**Keywords:** Anion gap, Lactate, Hyperlactatemia, Risk stratification, Systematic review, Meta-analysis

## Abstract

**Objective:**

This systematic review and meta-analysis seeks to determine the validity of the anion gap to screen for hyperlactatemia in critically ill patients. We have previously shown that the anion gap does not predict 31-day and in-hospital mortality in critically ill patients. The present review aims to add confirmatory evidence to identify whether the anion gap is a suitable tool for risk stratification in low-resource countries.

**Results:**

Nine studies reporting on 4504 samples from 2111 patients were included. The anion gap failed to detect hyperlactatemia defined as lactate above 2.5 mmol/l but showed good discriminatory ability for the detection of severe hyperlactatemia defined as lactate over 4 mmol/l. At the 2.5 mmol/l threshold, the anion gap had high specificity but low sensitivity for the detection of hyperlactatemia. A meta-analysis of correlation coefficients yielded high statistical heterogeneity. Therefore, in keeping with our previous findings, the use of the anion gap for risk stratification as an alternative to lactate cannot be recommended. However, the strength of the evidence we have synthesised is adversely affected by the small number of studies included, inconsistency of effect measures and positivity thresholds reported, and selection bias within individual studies. PROSPERO Registration Number: CRD42015016470 (registered on the 4th February 2015).

**Electronic supplementary material:**

The online version of this article (10.1186/s13104-017-2853-9) contains supplementary material, which is available to authorized users.

## Introduction

The anion gap (AG) reflects the concentration of unmeasured anions and is easily calculated from routine clinical chemistry analytes. Traditionally, the AG has been used as an alternative to lactate analysis; however, with the widespread availability of facilities for lactate analysis, the AG is now rarely used for this purpose in high-income countries. Nonetheless, in low-resource settings, where facilities for lactate analysis are frequently not available, the AG may have potential as a screening tool for hyperlactatemia in critically ill patients. Previous studies regarding the validity of AG as a screening tool for hyperlactatemia have yielded equivocal results [1, 2].

Our present review was intended to add confirmatory evidence to determine whether, in low-resource settings, efforts should be focused on making the best use of available resources to measure AG, or to widen access to lactate analysis. In a previous systematic review and meta-analysis, [3] we determined that the AG does not predict 31-day mortality, in-hospital mortality and comparable outcome measures. However, the findings of the previous review were limited by the poor methodological quality of included studies and significant statistical heterogeneity in meta-analysis. In the present review, we aimed to determine the validity of the observed and albumin-corrected AG to screen for hyperlactatemia in critically ill patients in a systematic review and meta-analysis.

## Main text

### Methods

This systematic review and meta-analysis adheres to the Preferred Reporting Items for Systematic reviews and Meta-analyses (PRISMA) standards [4]. A protocol was registered with PROSPERO, Registration Number CRD42015016470. Studies were eligible if the observed and/or albumin-corrected AG level was compared to arterial, venous or capillary lactate concentration in critically ill patients. Studies were excluded if the blood samples for AG and lactate were drawn more than 2 h apart. The search strategy, study selection and data extraction processes are described in our previous review [3] although in the current review no restriction on publication date was applied. Methodological quality was rated independently by two reviewers using a modified version of the Quality Assessment of Diagnostic Accuracy Studies (QUADAS-2) tool [5]. Agreement between reviewers was quantified using Cohen’s kappa and discrepancies were resolved by discussion.

Due to the inconsistency of effect measures and positivity thresholds reported by individual studies we have used a variety of statistical measures to summarise and synthesise the findings of included studies. Where a statistical synthesis would have been associated with substantial limitations we have opted for a narrative or graphical synthesis instead. Where statistical synthesis was possible, we used a random effects model where heterogeneity was high or moderate and a fixed and random effects model where heterogeneity was low. Heterogeneity was quantified using the I^2^ test. Forest plots displaying sensitivity and specificity were generated for studies reporting a common positivity threshold for both the AG and hyperlactatemia; a summary effect measure was not calculated. Likelihood ratios were calculated for each study. A Moses Littenberg summary ROC curve was generated for studies reporting a hyperlactatemia positivity threshold of 2.5 mmol/l [6]. Studies reporting sensitivity and specificity at a broad range of AG positivity threshold were selected so as to depict graphically the trade-off between sensitivity and specificity over a wide range of clinically relevant thresholds. A summary AUC value was not calculated. Studies reporting area under the ROC curve (AUC) for similar lactate thresholds were pooled in a generic inverse variance meta-analysis. Fisher’s Z-transformed Pearson product-moment correlation coefficients were also pooled in a generic inverse variance meta-analysis. Subgroup analysis was undertaken to assess whether heterogeneity in the meta-analysis of correlation coefficients could be explained by study setting or patient age. The summary ROC curve and the forest plots for sensitivity and specificity were generated in Review Manager 5.3 and correlation coefficients and AUCs were pooled in MedCalc version 15.4. All data are presented as effect estimates with 95% confidence intervals.

### Results

The results of the initial database search up to the commencement of full-text screening are described in our previous study [4] and in Additional file 1: Fig. S1. Sixteen articles were retrieved in full-text, of which two were excluded because the time between drawing samples for AG and lactate was not specified. A further five studies were excluded during data extraction: three studies were excluded because no relevant effect measure could be extracted, one study was excluded because only patients with metabolic acidosis were included and one study was excluded because only patients with lactate above 2.5 mmol/l were included. Therefore, nine studies were included in the systematic review (Additional file 2: Fig. S2). The characteristics of included studies are described in Additional file 3: Table S1. Five studies (56%) reported arterial lactate levels, one reported venous lactate levels, one reported both venous and arterial lactate levels and one did not specify the source. In all but two studies the samples for AG and lactate were drawn concomitantly or consecutively. In two studies a maximum time-frame of 30 min and 60 min respectively was given. Hyperlactatemia was defined as lactate above 2.5 mmol/l by six (67%) studies, as lactate above 4 mmol/l by one study (11%) and above 5 mmol/l by two studies (22%); the latter two thresholds will be referred to as severe hyperlactatemia here. The methodological quality of included studies is illustrated in Additional file 4: Fig. S3. Inter-rater agreement between reviewers was moderate (κ = 0.49), with differences in judgement mainly concerning the flow and timing domain. The patient selection and flow and timing domains were most frequently rated at risk of high or unclear bias.

**Fig. 1:**
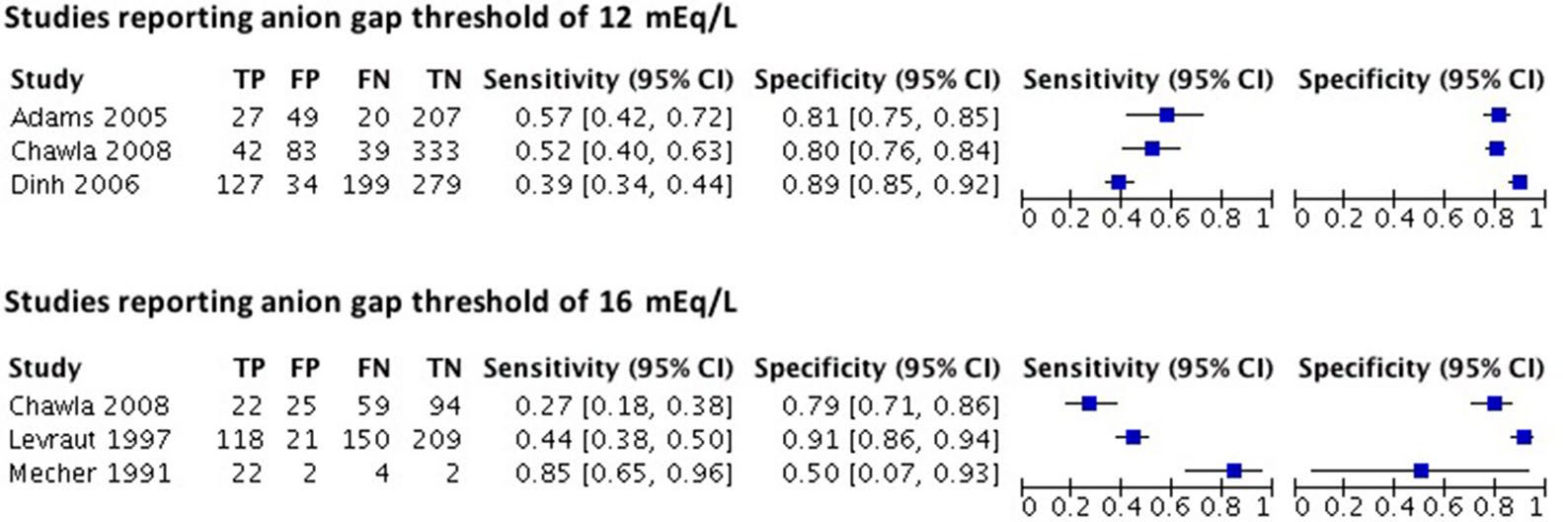
Forest plot of sensitivity and specificity of the observed AG at thresholds of 12 and 16 mEq/l to detect hyperlactataemia defined as lactate > 2.5 mmol/l (TP true positive, FP false positive, FN false negative, TN true negative, CI confidence interval)

Six studies reported sensitivity and specificity for a 2.5 mmol/L hyperlactatemia threshold, of which three studies reported an AG positivity threshold of 12 mEq/L and three studies reported an AG positivity threshold of 16 mEq/L (Fig. 1). For the former three studies, specificity was higher than 0.8 in all three studies but sensitivity was low with values ranging from 0.39 to 0.57. Results of the latter group appear heterogeneous with sensitivity values ranging from 0.27 to 0.85 and specificity ranging from 0.5 to 0.91. The positive likelihood ratios calculated for the above studies were all smaller than 10 and the negative likelihood ratios were larger than 0.1 and thus below the threshold recommended for clinical use. A Moses Littenberg summary ROC curve, including studies reporting AG positivity thresholds between 6 and 20 mEq/L, is shown in Additional file 5: Fig. S4. The trade-off between sensitivity and specificity appears to be poor. Judging from the magnitude of scatter of points among the predicted curve, there appears to be low to moderate heterogeneity.

**Fig. 2:**
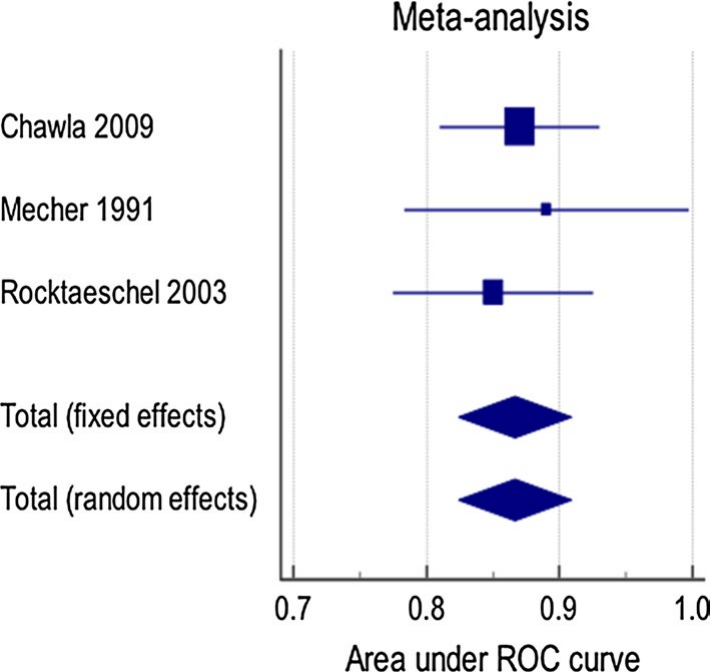
Forest plot showing random and fixed effects generic inverse variance meta-analyses for AUCs of the observed AG detecting severe hyperlactataemia defined as lactate > 4 mmol/l or 5 mmol/l, I^2^ = 0%

All three studies reporting AG in relation to severe hyperlactatemia reported AUCs, which were pooled in meta-analysis (Fig. 2); the summary AUC is 0.87 (0.82, 0.91) and no heterogeneity was observed (I^2^ = 0%).

**Fig. 3:**
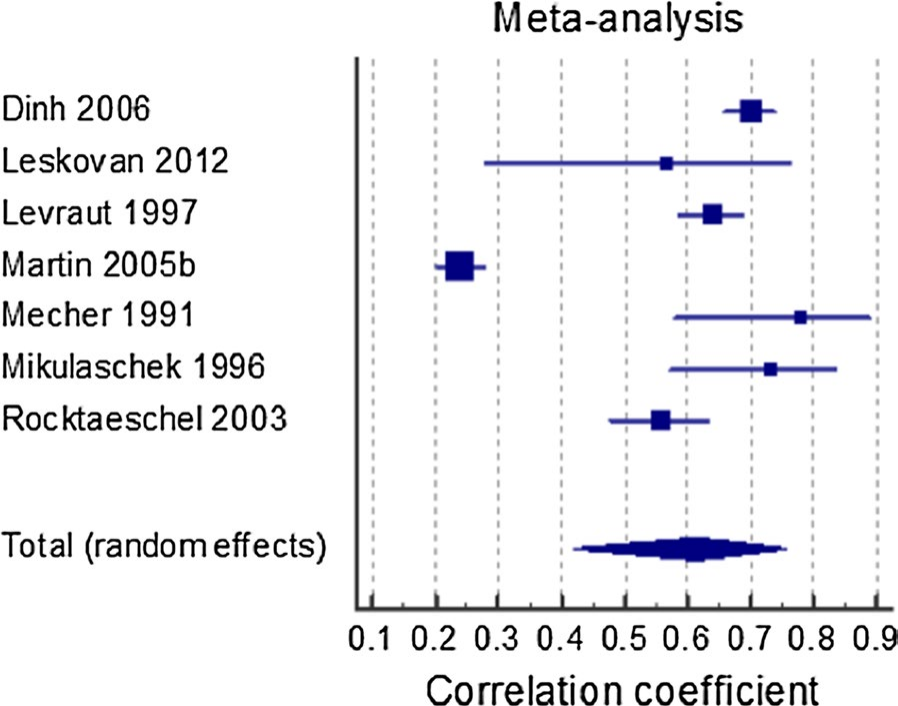
Forest plot for random effects generic inverse variance meta-analysis of correlation coefficients assessing the association between AG and lactate levels, I^2^ = 98%

Seven studies reported correlation coefficients; these were combined in meta-analysis (Fig. 3). In view of the high heterogeneity (I^2^ = 97.8%) the pooled effect estimate should not be interpreted. Most heterogeneity was due to the study by Martin 2005 [7]. Subgroup analysis on this meta-analysis demonstrated that neither study setting nor patient age influenced the summary correlation coefficient (p = 0.47 and 0.56 respectively), and heterogeneity remained high in all subgroups (data not shown).

Insufficient data were available for analysis of the validity of albumin-corrected AG.

### Discussion

The AG failed to detect hyperlactatemia defined as lactate above 2.5 mmol/l but showed good discriminatory ability for the detection of severe hyperlactatemia defined as lactate over 4 mmol/l. At the 2.5 mmol/l lactate threshold, the trade-off between sensitivity and specificity was poor and when sensitivity and specificity were analysed individually, the AG had high specificity but low sensitivity for the detection of hyperlactatemia. Importantly, a high hyperlactatemia threshold such as 4 mmol/l would miss patients at risk of adverse outcomes. Nichol and colleagues found that even patients with elevated lactate levels within the normal reference range are at higher risk of mortality compared to those with low lactate levels [8].

The poor screening power of the AG may be due to the variability in baseline AG levels between normal individuals. Where a patient’s baseline AG is low, an increment of up to 8 mEq/l may be necessary for the AG to fall outside the normal range [9]. Thus, a patient may become considerably hyperlactatemic before the AG positivity threshold is reached. Conversely, in patients with a high baseline AG, a small increase in lactate is sufficient to raise the AG above its positivity threshold. The ∆AG may be preferable but it was shown that in some patients, hyperlactatemia was not accompanied by a change in ∆AG [10].

Furthermore, the finding by Maciel and Park that lactate is only responsible for a minor percentage of metabolic acidosis may explain the poor screening power of the AG. In fact, unmeasured anions account for the majority of metabolic acidosis in critically ill intensive care unit patients [11]. Similarly, changes in albumin concentration are likely to be implicated in the poor screening power of the AG. The largest study included in this review [7] reported a larger correlation between albumin-corrected AG and lactate compared to the uncorrected AG. However, other studies found that correcting the AG for albumin did not improve the detection of hyperlactatemia [12].

The high heterogeneity in the meta-analysis of correlation coefficients remains largely unexplained. Subgroup analysis was undertaken to determine whether patient age or clinical setting influence the pooled effect estimate but too few studies were available to conduct subgroup analysis on further factors. Other factors, which may explain heterogeneity include the type of clinical chemistry analysis, differences between arterial, venous and capillary lactate and baseline albumin level.

Overall, the studies included in the present review are of higher quality than those included in our previous review, in which we investigated the ability of the AG to predict 31-day or in-hospital mortality [3]. The main reason may be that fewer variables need to be accounted for to establish the relationship between AG and lactate whereas mortality is affected by multiple factors, which can be difficult to control for.

Taken together, the results of the present and previous reviews suggest that the AG should not be recommended for risk stratification. Instead, we believe that future research should focus on widening access to lactate analysis, for example in the form of hand-held point-of-care devices. A study of septic patients admitted to the emergency department found that point-of-care lactate devices reduced the time to administration of intravenous fluids, intensive care unit admission and mortality [13]. Point-of-care testing was found to improve health outcomes in low-income countries [14] and the authors felt that introducing point-of-care testing for lactate in a tertiary obstetric unit in Malawi was feasible and well received by staff [15].

### Conclusion

The AG has low sensitivity to detect hyperlactatemia at a clinically relevant positivity threshold of 2.5 mmol/l but has good discriminatory ability for the detection of severe hyperlactatemia defined as lactate over 4 mmol/l. In keeping with the findings of our previous study on the validity of the AG to predict mortality in critically ill patients, the use of the AG as an alternative to lactate measurement cannot be recommended.

## Limitations

The main limitation of this review is the small number of studies included and inconsistency of effect measures and AG positivity thresholds reported by individual studies. Several studies failed to report sensitivity and specificity or failed to report the prevalence of hyperlactatemia in the study population for all lactate positivity thresholds examined. Furthermore, some studies did not enrol a consecutive or random sample of patients and failed to specify whether data for all patients was available for analysis, which may have led to sampling bias. Whilst in most studies the samples for AG and lactate were drawn concomitantly, in two studies a maximum time-frame of 30 and 60 min was given respectively. One study [16] reported that whilst it was standard practice to draw the samples simultaneously, there may have been exceptions. Interventions and treatment during resuscitations in critically ill patients may result in changes to the physiology that make such results unreliable. Lastly, two studies had to be excluded because the time between the measurement of AG and lactate was not specified.

The review methodology was limited by its language restriction to articles published in English, German or French, which may have introduced publication bias. Furthermore, the study selection process was undertaken by a single reviewer, which may have increased the risk of missing relevant studies.

## Additional files

**Additional file 1: Fig. S1.** Flow chart of search and selection process of our previous study on the ability of the anion gap to predict 31-day and in-hospital mortality.

**Additional file 2: Fig. S2.** Flow chart of the search and selection process of the present study.

**Additional file 3: Table S1.** Characteristics of included studies. HL (hyperlactatemia), ICU (intensive care unit), NA (data not available).

**Additional file 4: Fig. S3.** Risk of bias and applicability concerns graph: reviewers’ judgements on quality domains relevant to diagnostic accuracy studies for each included study.

**Additional file 5: Fig. S4.** Moses Littenberg-based summary ROC curve for the ability of observed AG to detect hyperlactataemia defined as lactate > 2.5 mmol/l.

## Abbreviations

AG: anion gap
AUC: area under ROC curve
PRISMA: Preferred Reporting Items for Systematic Reviews and Meta-analyses
QUADAS: Quality Assessment of Diagnostic Accuracy Studies
ROC: receiver operator characteristics
