# Supplementary figures and images for: A systematic review and diagnostic test accuracy meta-analysis of the validity of anion gap as a screening tool for hyperlactatemia
Source: BMC Res Notes. 2017 Nov 3;10:556. doi: 10.1186/s13104-017-2853-9 (PMC5670505; doi:10.1186/s13104-017-2853-9)

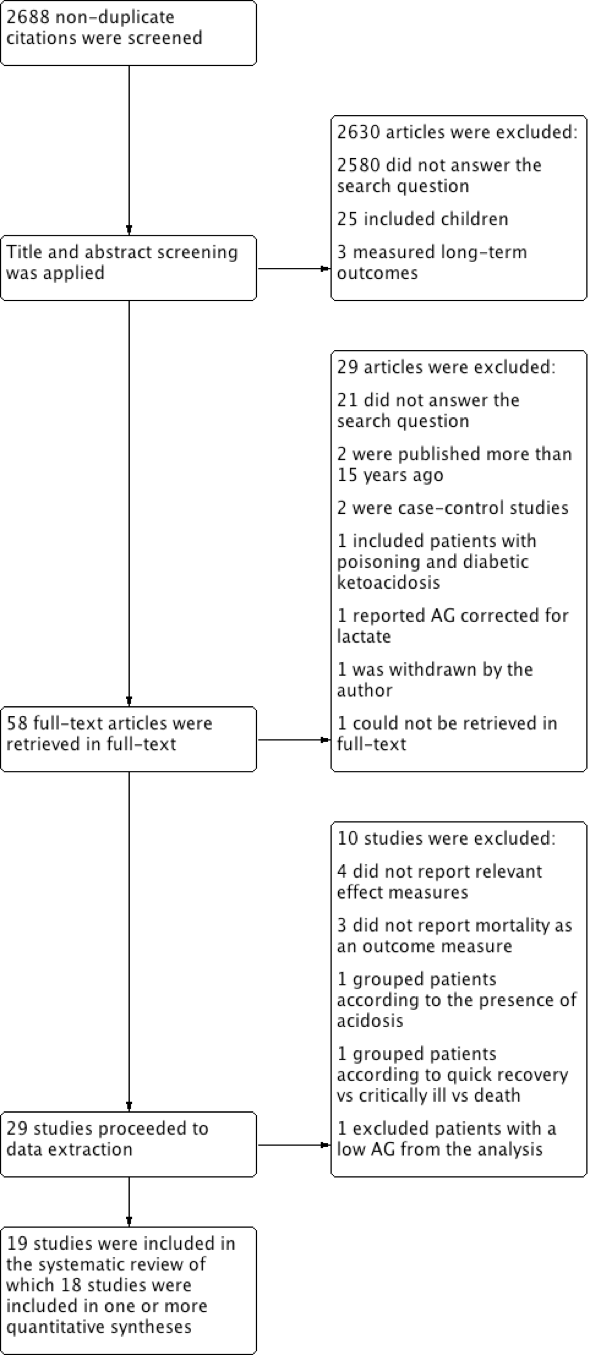

Supplement: Supplementary file 1 — Additional file 1: Fig. S1. Flow chart of search and selection process of our previous study on the ability of the anion gap to predict 31-day and in-hospital mortality. [file 13104_2017_2853_MOESM1_ESM.png]

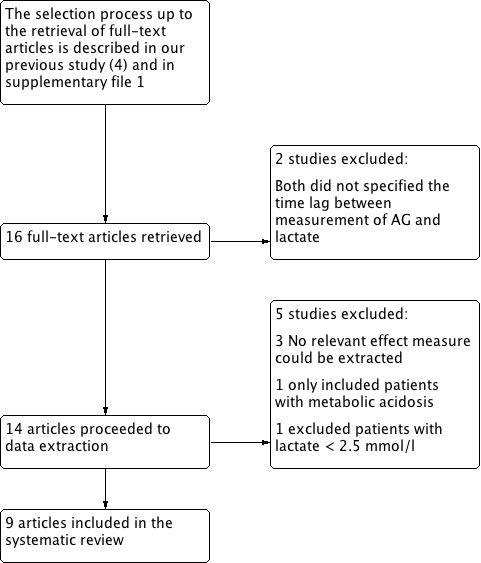

Supplement: Supplementary file 2 — Additional file 2: Fig. S2. Flow chart of the search and selection process of the present study. [file 13104_2017_2853_MOESM2_ESM.png]

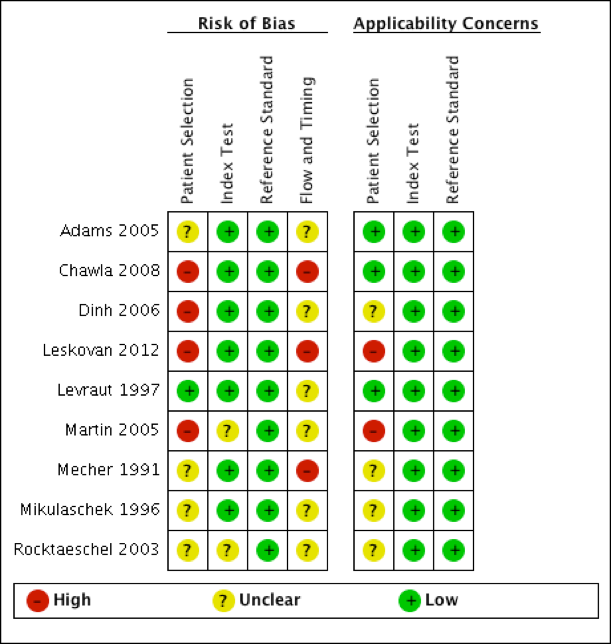

Supplement: Supplementary file 4 — Additional file 4: Fig. S3. Risk of bias and applicability concerns graph: reviewers’ judgements on quality domains relevant to diagnostic accuracy studies for each included study. [file 13104_2017_2853_MOESM4_ESM.png]

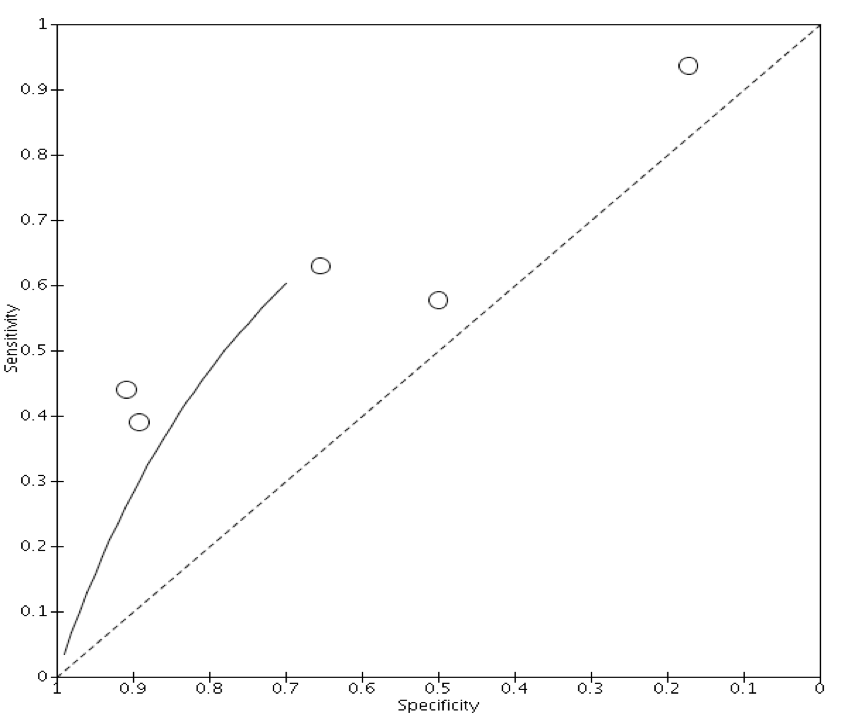

Supplement: Supplementary file 5 — Additional file 5: Fig. S4. Moses Littenberg-based summary ROC curve for the ability of observed AG to detect hyperlactataemia defined as lactate > 2.5 mmol/l. [file 13104_2017_2853_MOESM5_ESM.png]
