# Supplementary material for: A systematic review and diagnostic test accuracy meta-analysis of the validity of anion gap as a screening tool for hyperlactatemia
Source: BMC Res Notes. 2017 Nov 3;10:556. doi: 10.1186/s13104-017-2853-9 (PMC5670505; doi:10.1186/s13104-017-2853-9)
Supplement: Supplementary file 3 — Additional file 3: Table S1. Characteristics of included studies. HL (hyperlactatemia), ICU (intensive care unit), NA (data not available). [file 13104_2017_2853_MOESM3_ESM.docx]

**Characteristics of included studies**

| 1st author and reference | Nr | Year | Country | Study population | Study design | Nr of  samples  (patients) | Mean  age | AG  thresholds  /mEq/L | Lactate thresholds  /mmol/l | Prevalence HL in study population  % | Type of lactate |
| --- | --- | --- | --- | --- | --- | --- | --- | --- | --- | --- | --- |
| Adams [2] | 1 | 2006 | US | Emergency admissions | Retro-spective | 303  (272) | 68 | 6; 12 | 2.5 | 16 | Venous, arterial |
| Chawla [16] | 2 | 2008 | US | ICU patients | Retro-spective | 497  (143) | 62 | 10; 12; 14; 16 | 2.5; 4 | 16 | arterial |
| Dinh [12] | 3 | 2006 | US | All hospital patients | Retro-spective | 639  (356) | NA | 12 | 2.5 | 51 | venous |
| Levraut [[17](#_ENREF_14)] | 4 | 1997 | France | ICU patients | Pro-spective | 498 | 52 | 16 | 2.5; 5; 10 | 54, 22, 6 | arterial |
| Rocktaeschel [1] | 5 | 2003 | Australia | ICU patients | Retro-spective | 300 | median 65 | NA | 5 | NA | arterial |
| Mecher [18] | 6 | 1991 | US | Severe sepsis/ ICU | Pro-spective | 30 | 73 | 16; 20 | 2.5; 5; 10 | 87, 47, 17 | arterial |
| Leskovan [19] | 7 | 2012 | US | Trauma patients | Retro-spective | 33 | 63 | NA | NA | NA | NA |
| Martin [7] | 8 | 2005 | US | Trauma patients | Retro-spective | 2152  (427) | 38 | 17 | 2 | 18 | arterial |
| Mikulaschek [20] | 9 | 1996 | US | Trauma patients | Retro-spective | 52 | 36 | 16 | 2.5 | NA | NA |

*Table S1. Characteristics of included studies. HL (hyperlactatemia), ICU (intensive care unit), NA (data not available).*

Note: references 17-20 are not cited in the main manuscript so are listed here:

[17]: Levraut J, Bounatirou T, Ichai C, Ciais JF, Jambou P, Hechema R, et al. Reliability of anion gap as an indicator of blood lactate in critically ill patients. Intensive care medicine. 1997;23(4):417-22.

[18]: Mecher C, Rackow EC, Astiz ME, Weil MH. Unaccounted for anion in metabolic acidosis during severe sepsis in humans. Critical Care Medicine 1991;19(5):705-11.

[19]: Leskovan JJ, Justiniano CF, Bach JA, Cook CH, Lindsey DE, Eiferman DS, et al. Anion gap as a predictor of trauma outcomes in the older trauma population: correlations with injury severity and mortality. The American Surgeon 2013;79(11):1203-6.

[20]: Mikulaschek A, Henry SM, Donovan R, Scalea TM. Serum lactate is not predicted by anion gap or base excess after trauma resuscitation. The Journal of Trauma 1996;40(2):218-22
